# Interventions to foster family inclusion in nursing homes for people with dementia: a systematic review

**DOI:** 10.1186/s12877-020-01836-w

**Published:** 2020-10-30

**Authors:** Ramona Backhaus, Linda J. M. Hoek, Erica de Vries, Jolanda C. M. van Haastregt, Jan P. H. Hamers, Hilde Verbeek

**Affiliations:** Care and Public Health Research Institute (CAPHRI), Department of Health Services Research, Maastricht University, P.O. Box 616, 6200 MD Maastricht, The Netherlands

**Keywords:** Family involvement, Long-term care, Psychogeriatrics

## Abstract

**Background:**

Family inclusion in nursing homes is central to the provision of individualized care for people with dementia. Although positive effects can be recognized, barriers have been identified that hamper family inclusion in nursing homes. Specifically for people with dementia, insight into the content of interventions to foster family inclusion is lacking.

**Methods:**

A systematic review was performed by systematically searching the databases PubMed, Cinahl, PsycInfo and Embase. Studies were eligible if they examined (1) nursing home settings, (2) interventions to foster the inclusion of family members from people with dementia, (3) were original research articles in which effects/experiences of/with these interventions were evaluated, and (4) were written in English, Dutch or German. Findings were summarized systematically.

**Results:**

Twenty-nine studies were included. Two interventions were targeted at creating family-staff partnerships from a two-way perspective. Other interventions focused on single components, such as including family members in formal decisions (*n* = 9), enabling them to make better informed decisions and/or participate more actively (*n* = 7), or providing psychoeducation for family members (*n* = 3). Within the interventions, family and staff members are often treated differently. Effects on actual increase in family inclusion remain unclear.

**Conclusions:**

Very few interventions exist that try to enhance equal family-staff partnerships in nursing homes. Future interventions should pay specific attention to mutual exchange and reciprocity between family and staff. As little is known about promising (components of) interventions to foster family inclusion in nursing homes for people with dementia, more effectiveness research is needed.

## Background

Person-centered care approaches are increasingly being implemented in various healthcare settings and are widely recognized as an essential component of quality care [1, 2]. While, ideally, these approaches should involve the person (i.e., the patient/resident), their families (not only the primary caregiver, but all family members (e.g., partners, (grand) children, siblings)), and the care providers, a recent review of systematic reviews has demonstrated that only a minority of person-centered care interventions pay attention to the role of family members [1]. In nursing homes, family inclusion is a central element for the provision of individualized care for people with dementia [3]. As the relationship between people with dementia and their family members has developed over a life course, it is likely that families’ roles continue once a person with dementia enters a nursing home [4–6]. Early research indicated that it is a myth to believe that family members abandon their relatives that live in a nursing home [7, 8]. In contrast, although their role might change in the sense that they are less involved in the physical care and decision-making [9], they stay involved after the person with dementia moves into a nursing home. Family members’ sharing of information on the person’s biography, meaningful activities and daily life preferences with staff might lead to positive effects for nursing home residents [10]. In addition to people with dementia, family members themselves may also benefit from the inclusion of family in nursing home settings. Family inclusion refers to creating democratic engagement of families within nursing homes, by providing family members with opportunities and resources that empower them to actively participate in their relatives’ life as well as in the nursing home as a community [6]. For family members, being involved in the nursing home may lead to enhanced satisfaction with the care provided to residents and an enriched own wellbeing [11, 12]. At the same time, family inclusion has a potential for conflicts, as different family members (e.g., a son and a daughter of the resident) may differ in their opinion on ‘what is best’ for the resident. As family members provide help to people with dementia in various ways, e.g., through providing hand-on or socio-emotional care or monitoring [13], staff members may also benefit from their involvement. In addition, nursing home organizations as a whole may benefit from the inclusion of family members. From a business perspective, family members in nursing homes are indirect customers who do not purchase services themselves, but only accompany the direct customer [14]. Nevertheless, research has indicated that nursing home organizations may benefit from getting their indirect customers more engaged with the organization [15]. Organizations in different sectors increasingly recognize the importance of (indirect) customer engagement behaviors [15, 16]. These behaviors can be defined as customers’ voluntary, helpful behaviors towards an organization after and beyond purchasing goods or services [14]. Indirect customer engagement behaviors of family members in nursing homes can occur in interacting with staff members, providing feedback or complying with organizational rules and procedures, but also in the interaction with other (potential, indirect) customers, by helping them or by spreading a positive word of mouth or writing online reviews about the nursing home [14].

Although positive effects of family inclusion for residents, family members, staff and the organization as a whole can be recognized, a wide variety of barriers have been identified that hamper family inclusion in nursing homes. In a sociopolitical environment of staff shortages and scarce resources in nursing homes, family members are easily seen as a commodity [6, 17] or as a resource for augmenting staff [18]. While family members in many countries are considered as being able to form an unpaid workforce in nursing homes, adequate support of family members in nursing homes is often lacking [6]. For example, family members are often left alone to deal with complex emotions related to the decision that a loved one has to enter a nursing home [6, 19]. Staff members often find it difficult to collaborate with family members and may consider them as being ‘difficult’ or demanding [20, 21]. While family members need to understand that there are limits to what a nursing home can offer, it is important that nursing home organizations take into account family members’ capacities and personal situation too. Professional caregivers could see family members as a valuable resource in providing person-centred care for their residents, instead of fearing that family may become additional clients themselves and increase their workload. Contextual factors like the geographic proximity, the employment status or family members’ own health status may have an impact on the role family members want to or can play in nursing homes [6]. In addition, Cohen et al. [11] demonstrated that family involvement for residents with dementia is different compared to residents without dementia. They found that while family members of people with dementia spent more time on activities to support resident care (e.g., related to nutrition, mobility or discussing care with staff), family members of residents without dementia spent more time on their social and community engagement (e.g., taking residents on trips, shopping), often outside the nursing home. This demonstrates that, particularly for family members of people with dementia, the tasks often go beyond solely visiting the relative. Instead, family members conduct caregiving tasks that could be considered a staff responsibility, which might be particularly burdensome for family members [11].

In a recent critical examination of how resident care is negotiated among staff and family members in nursing homes, Puurveen et al. [22] conclude that in formal care conferences (also known as case conferences or inter−/multidisciplinary team meetings), family members occupy a ‘marginal position relative to staff.’ Instead of being a dialogic space promoting family inclusion, they found that care conferences are spaces in which staff members perform ‘expert one-way communication’ [22]. Considering the citizen participation ladder of Edelenbos & Klijn [23], five levels of participation can be distinguished, i.e., ‘informing,’ ‘consulting,’ ‘advising,’ ‘co-producing’ and ‘co-deciding.’ Translated to family inclusion in nursing homes, ‘informing’ means that the nursing home organization determines the agenda for decision-making and informs family about decisions only, while ‘co-deciding’ stands for equal shared decision-making between family and staff members. Thus, the degree and type of family inclusion in the care conferences assessed by Puurveen et al. [22] can be classified as ‘informing’ only. To meaningfully contribute to person-centered care, especially for people with dementia, formal care conferences and other interventions aimed at increasing person-centeredness through family involvement should promote mutual exchange and reciprocity between staff and family members and should empower family members to participate as equals [22, 24].

In earlier systematic reviews, Haesler et al. [25–28] assessed factors that are important in the development of constructive family−staff relationships in the care of older institutionalized adults, including both hospitals and institutional long-term care settings. Based on their most recent review conducted in 2010 [27], they conclude that interventions to promote constructive family−staff relationships were those that include collaboration in care planning and decision-making, promote effective communication skills, define a clear process and involve multidisciplinary healthcare teams. However, as the engagement of family members of people with dementia differs from that of family members of residents without dementia, the opportunities and resources that empower them to actively participate in their relatives’ life as well as in the nursing home as a community might also differ. Specifically for people with dementia in nursing homes, an overview of interventions to foster the inclusion of family members of people with dementia is lacking. A qualitative meta-synthesis conducted by Petriwskyj et al. [29] provides insight into the experiences of family involvement in decision-making for people with dementia in residential care, without considering specific interventions. In 2015, Nguyen et al. [30] published a protocol for a systematic review on interventions to improve communication and cooperation in order to promote effective family−staff relationships for family members of people with dementia living in residential aged care facilities.

The aim of this systematic review is to obtain insight into the content of interventions to foster the inclusion of family members (e.g., partners, (grand) children, siblings) of people with dementia living in nursing homes within the nursing home setting. We take a broad view and do not only focus on interventions that contribute to family inclusion in family−staff interactions, but also take into account interventions to facilitate family−resident or family−family engagement in the nursing home setting. This means that, besides focusing on family−staff relationships, we also pay attention to how to enable family members to better engage with their relatives or with family members of other residents, and therefore consider interventions that contribute to family involvement and give a voice to family within the whole nursing home community. We are predominantly interested in the content of existing interventions, with the aim of contributing to the development of future interventions to involve family members of people with dementia within the nursing home setting.

## Methods

### Search strategy

A systematic review was performed by systematically searching the databases PubMed, Cinahl, PsycInfo and Embase. The search strategy included terms related to family, dementia, nursing homes, involvement, and interventions (see Table 1). Search terms with respect to family (e.g., family OR caregiver* OR spous* OR relativ* OR informal care*) were combined with the Boolean operator AND with search terms related to dementia (dementia OR demented OR Alzheimer* OR cognitive declin*), search terms related to the nursing home setting (long-term care OR care hom* OR housing for the elderly OR special care unit* OR homes for the aged OR institutional*), search terms related to involvement (participat* OR relationship* OR collaborat* OR involve* OR interact* OR role* OR engag*), and search terms related to interventions (program* OR intervention* OR strateg*). Articles published between January 1st, 2000 and February 7th, 2019 were included. Bibliographies of included articles were searched for additional references.

**Table 1:** Steps and detailed search terms used in the PubMed search*

Step	Search terms
**1**	***Subject area 1: Family members***
	(((((((family[MeSH Terms]) OR caregivers[MeSH Terms]) OR spouses[MeSH Terms]) OR family[Title/Abstract]) OR relativ*[Title/Abstract]) OR spous*[Title/Abstract]) OR caregiver*[Title/Abstract]) OR (informal care*[Title/Abstract])
**2**	***Subject area 2: Dementia***
	((((dementia[MeSH Terms]) OR dementia[Title/Abstract]) OR demented[Title/Abstract]) OR alzheimer*[Title/Abstract]) OR (cognitive declin*[Title/Abstract])
**3**	***Subject area 3: Nursing homes***
	((((((((((long-term care[MeSH Terms]) OR residential facilities[MeSH Terms]) OR (nursing hom*[Title/Abstract])) OR (residential care[Title/Abstract])) OR (assisted living[Title/Abstract])) OR (long-term care[Title/Abstract])) OR (care hom*[Title/Abstract])) OR (housing for the elderly[Title/Abstract])) OR (special care unit*[Title/Abstract])) OR (homes for the aged[Title/Abstract])) OR institutional*[Title/Abstract]
**4**	***Subject area 4: Involvement***
	((((((participat*[Title/Abstract]) OR relationship*[Title/Abstract]) OR collaborat*[Title/Abstract]) OR involve*[Title/Abstract]) OR interact*[Title/Abstract]) OR role*[Title/Abstract]) OR engag*[Title/Abstract]
**5**	***Subject area 5: Interventions***
	((program*[Title/Abstract]) OR intervention*[Title/Abstract]) OR strateg*[Title/Abstract]

* Detailed search strategies used in the other databases (Cinahl, PsycInfo and Embase) are available upon request

### Eligibility criteria

Studies were eligible if they (1) examined specifically the nursing home setting, (2) examined interventions to foster the inclusion of family members from people with dementia, (3) were original research articles in which the effects/experiences of/with these interventions were evaluated, and (4) were written in English, Dutch or German.

### Study screening and data extraction

Retrieved articles were managed in an Endnote library (version X8). Two researchers (RB, LJMH) independently screened the titles of all articles for relevance. After reaching consensus on the result of independently screening the titles, both researchers screened the abstracts of potentially relevant articles. After reaching consensus on the result of independently screening the abstracts, full-text articles were obtained for all potentially relevant studies. Both researchers independently screened the full-text articles and scored them as ‘include,’ ‘possibly include’ or ‘exclude.’ By discussing disagreement between the two researchers, consensus about the final list of included studies was reached. The principal researcher developed a standardized data extraction form (specifically developed for the current study) and extracted data from included articles. All extracted data were double-checked by a second researcher (EdV). For each article, data on the following aspects were extracted: author; publication year; country; sample characteristics; research methods; aim of the study; components and duration of interventions; treatment of control group; relevant outcome measures/themes analyzed; and study findings. All extracted data were discussed within the research team.

### Data synthesis and analysis

Because of the heterogeneity in studies regarding their design and content, and as we were predominantly interested in the components of interventions, no meta-analysis or quality assessments were conducted. Instead, the findings of included studies were summarized systematically, based on the aim and type of the intervention and its individual components, by two members of the research team (RB, EdV) and discussed within the research team. First, we distinguished between interventions that facilitated family inclusion in family−staff interactions and those that facilitated family−resident or family−family engagement. Second, based on the content of the included studies, the interventions facilitating family inclusion in family−staff interactions were further classified into interventions aimed at (1) creating family−staff partnerships, (2) including family members in formal decisions, (3) enabling them to make better informed decisions and/or participate more actively in future interactions with staff, or (4) providing psychoeducation for family members to, inter alia (i.a., meaning ‘among other things’), improving future interactions with staff. For each intervention, a summary of its effects was written by two members of the research team and discussed within the research team.

## Results

**Fig. 1:**
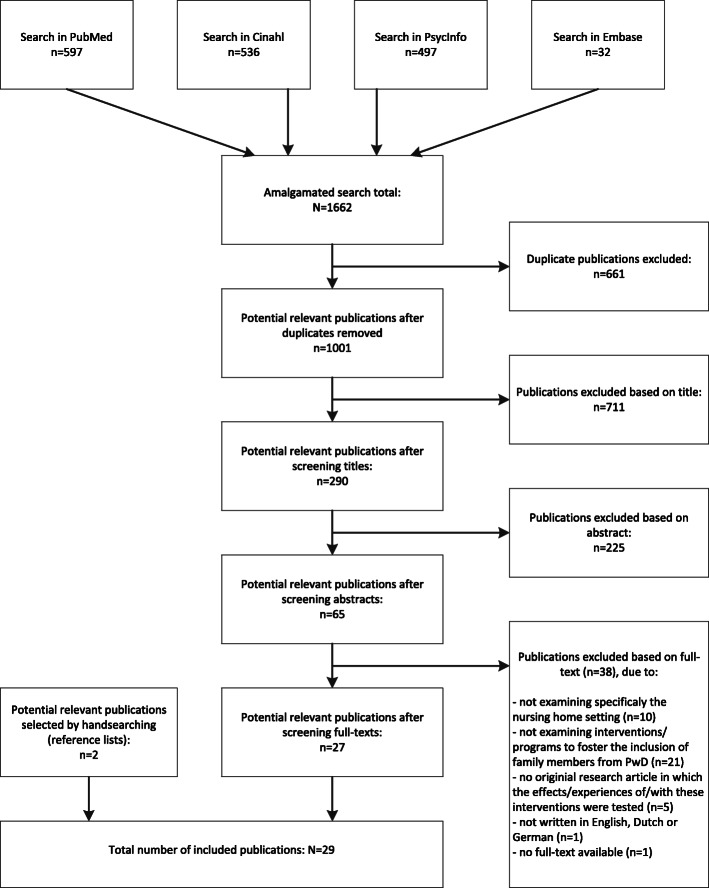
PRISMA flow diagram of the search process

Figure 1 presents the PRISMA flow diagram of the inclusion process. In total, 29 studies were included (Table 2).

**Table 2:** General characteristics of included studies (*n* = 29)

Author	Country	Type of intervention	Effects/Results
Agar et al. (2017) [31]	Australia	Facilitated family case conferencing	No significant group effect for family members’ satisfaction with care during the last 90 days of residents’ life.
Ampe et al. (2017) [32]	Belgium	‘We DECide’ – Disussing End-of-Life Choices	Advance care planning policy was significantly more compliant with ‘best practice’ after the intervention, while policy in the control group was not. Advance care planning discussions did not take place more often, nor were residents and family more involved in the discussions.
Arcand et al. (2009) [33]	Canada	Pilot educational program for nursing staff and physicians on comfort care and advanced dementia; booklet	No significant effects of the intervention were found, although the post-intervention group expressed greater satisfaction in the area of communication with the healthcare team (8.0 vs. 6.6) and greater satisfaction with end-of-life care (8.3 vs. 7.3).
Beer et al. (2011) [34]	Australia	Educational intervention for GPs and care staff	Nearly two third of general practitioners reported that the participants’ learning needs were met ‘entirely’. 95% of the staff members reported that the session met participants’ learning needs. In addition, qualitative feedback was very positive.
Bramble et al. (2011) [35]	Australia	Family involvement in care (FIC) intervention	Participation in FIC led to an improvement of caregiver knowledge. Knowledge about dementia increased significantly. Family satisfaction was negatively related to staff consideration of relatives and management effectiveness. Staff well-being and job satisfaction were negatively related to their perceived inappropriate behavior of residents.
Brazil et al. (2018) [36]	UK	Family focused advance care planning (ACP) intervention	Reduction in total Decisional Conflict Scale score in the intervention group compared with the usual care group.
Chisholm et al. (2018) [37]	US	Goals of Care video decision aid for families of residents with advanced dementia	Staff perceived the intervention as positive and it was perceived as ‘more compatible with current practices’ by male staff, nurses, more experienced staff.
Dassa (2018) [38]	Israel	Individualized database using personal music and photos that present life episodes	All three participants encountered difficulties when visiting the nursing home, mostly related to communication barriers. All three reported that using the individualized database led, for example, to better communication with their residents and less feelings of disconnection.
Davis et al. (2011) [39]	US	Family Intervention: Telephone Tracking-Nursing Home (FITT-NH) for improving dementia caregivers’ adjustment following nursing home placement	Caregivers receiving FITT-NH showed a greater reduction in feelings of guilt related to placement compared to standard care. And also reported fewer problems and concerns with nursing home care (hassles) compared to standard care. FITT-NH caregivers also showed a trend toward a resumption of pre-caregiver activities (% of previous activities) compared to standard care.
Ducharme et al. (2005) [40]	Canada	‘Taking Care of Myself’, psychoeducational group program for daughters	Daughters’ competence in dealing with staff and their perceived challenge of the caregiver role increased.
Jablonski et al. (2005) [41]	US	Family involvement in care (FIC) intervention	The experimental group showed less global deterioration during the study. Increased family involvement showed less global deterioration in residents.
Kellett et al. (2010) [42]	Australia	Family Biography Workshop (FBW)	Participating family members were enabled to ‘stand outside’ and see the residents as a whole person. For staff, it was helpful to see the resident in a ‘family context’. Residents benefited, as staff’s know how on engaging with them increased.
Kuhn & Forrest. (2012) [43]	US	Pilot palliative care education, training, consultations, and administrative coaching	Limited improvements for residents, staff and family members were demonstrated at the first nursing home (site 1), and significant improvements at the other nursing home (site 2).
Maas et al. (2004) [44]	US	Family Involvement in Care (FIC) partnership intervention	An intervention effect was found for family disregard with staff, and the effect varied by generation. For conflicts with staff, no significant effect was found. An intervention effect was found for families’ perceptions of physical care and activities: evaluation of physical care significantly increased for the intervention group and the intervention effect for activities was conditioned by interaction with generation (again, the trend for the older comparison group was significantly negative. No intervention effect on families’ perception of management effectiveness was found.
Mariani et al. (2018) [45]	Italy Netherlands	Staff training program on the use of shared-decision making (SDM) with residents and family caregivers in the care planning process	Many care plans developed during the intervention showed a high level of agreement with the care planning recommendations. In Italian and Dutch problem statements, the problem statements became clearer. In Italy, documentation of objectives and residents’ and families’ involvement increased, too.
McNiel & Westphal (2018) [46]	US	Namaste Care program	Results suggest that Namaste Care™ may be useful for residents who can no longer participate in ‘regular’ nursing home activities.
Moore et al. (2017) [47]	UK	‘Compassion Intervention’ to enhance end-of-life care in advanced dementia	Due to the recruitment of only four family members, the researchers were unable to assess the effects on family members.
Paun et al. (2015) [48]	US	Chronic Grief Management Intervention (CGMI)	Overall, no significant effects. At baseline, family members in the intervention group scored higher and therefore differed significantly from the control group with regard to loss of relationship with their family member placed in long-term care and heartfelt sadness and longing). Family members in the intervention group were highly satisfied with the program.
Reinhardt et al. (2014) [49]	US	Discussing and providing information about end-of-life care options with family members and psychosocial support for family members	No significant effects for depressive symptoms or life satisfaction of family members.
Robison et al. (2007) [50]	US	Partners in Caregiving in the Special Care Unit Environment (PIC-SCU)	In the intervention group, staff behavior (providing news, encouragement or suggestion to family) increased over 6 months, but did not change significantly for the control group. In the intervention group, families’ ease of talking to staff increased and remained elevated at 6 months, whereas scores for the control group were static. When examining intergenerational differences between spouses and other some generation family members and children or younger generation family members, same-generation family members in the intervention group increases their involvement in the short term, whereas control family involvement declined (no significant differences for younger generation family members). All other outcomes were not statistically significant.
Rosemond et al. (2017) [51]	US	Goals of Care intervention: video decision-aid about goals of care viewed by family members of residents with dementia, followed by their participation in a care plan discussion with staff	Deciding on goals of care in the presence of trust vs absence of trust: (When decision makers expressed trust in the NH, positive relationships with staff were evident): 1. End-of-life experience was positive vs negative 2. Goals of care discussions were dynamic vs perceived to be ignored 3. Formal goals of care discussions were not always necessary vs created confusion.
Rosen et al. (2003) [52]	US	Web-based educational program (prototype)	Comparing answers before and after the program, family members gained knowledge about dementia. Most respondents indicated that the program was ‘very much’ providing information he/she needed, 2 indicated that ‘somewhat’. All indicated that the program would be ‘very much’ helpful to family members who recently placed a loved one in a nursing home.
Saini et al. (2016) [53]	UK	‘Compassion Intervention’ to enhance end-of-life care in advanced dementia	Four major themes described strategies for improving practice: family and staff education about dementia progression and end–of-life care; appreciating in-depth end-of-life discussions compared with simple documentation of care preferences; provision of time and space for sensitive discussions; and having an independent healthcare professional or team for the end-of-life discussions.
Snyder et al. (2013) [54]	US	Decision aid to improve decision making about feeding options in dementia care	Comparing answers before and after exposure to the decision aid, family members had/were after exposure: more correct answers to knowledge items on tube feeding, decreased expectations of benefits from tube feeding, decreased levels of overall decisional conflict, unchanged treatment preferences (nearly all chose assistance with oral feeding over tube feeding and preferences did not change, more certain about their choice of oral feeding.
Stacpoole et al. (2017) [55]	UK	Namaste Care program	Reaching out to each other: 1. Families re-connected with their relatives 2. Families recognized the compassion of care staff and appreciated the difficulties of caring for people with advanced dementia; Enhanced well-being: 1. For one wife, the guilt she felt for placing her husband in the care home and leaving him each day after visiting was lifted 2. A daughter summarized that Namaste made visits easier, helped her re-connect with her mother and recover a meaningful role in her mother’s life
Stirling et al. (2014) [56]	Australia	Tool to aid talking about dementia and dying	The tool facilitated a more open dialogue between the palliation resource nurses (a role specifically developed) and family members. These nurses as well as family members gained confidence in discussing the death of their relative with dementia. In some cases, specific decisions around future care were discussed. Family members and nurses were satisfied with these discussions.
Van der Steen et al. (2012) [57]	Canada Netherlands Italy	Family booklet on comfort care in dementia	The booklet was found highly acceptable and useful by Canadian and Dutch families, but less so by Italian families.
Van der Steen et al. (2011) [58]	Netherlands	Family booklet on comfort care in dementia	There was a great need for written explanation about palliative care in dementia. The booklet is seen as suitable and useful. All respondents saw a role for caregivers in giving out the booklet. Half of the respondents felt that the booklet should also be available without having contact with caregivers; sometimes even before admission. An adapted version might support caregivers and family in practice.
Verreault et al. (2018) [59]	Canada	Multicomponent intervention to improve quality of care and quality of dying in advanced dementia	In the intervention group, compared to usual care: more families received an information booklet; more families had contact with the physician in last month of life; more families had face-to-face contact with the physician in last month of life; no difference in number of families that had a discussion of advanced directives in last month of life; more frequent use of volunteers to give family a respite.

### General characteristics of included studies

Most of the studies (*n* = 25) aimed to contribute to family inclusion in family−staff interactions, while only a few (*n* = 6) considered interventions contributing to family−resident or family−family engagement. Two of the studies [40, 42] aimed to cover both family−staff interactions and family−resident engagement. The 29 included studies were published from 2003 onwards, with more than half of them published within the last 5 years. Eleven of the included studies were published in 2017 or 2018. Studies were conducted in Australia (*n* = 5), Belgium (*n* = 1), Canada (*n* = 4), Israel (*n* = 1), Italy (*n* = 2), the Netherlands (*n* = 3), the UK (*n* = 4), and the US (*n* = 12). In total, 24 different interventions were tested in the included studies. In two studies [57, 58], the same data set and findings were presented.

### Components of interventions to foster family−staff interactions

We identified 25 studies in which 21 different interventions for fostering family inclusion in family−staff interactions were tested (Table 3). These 21 interventions were aimed at creating family−staff partnerships (*n* = 2), including family members in formal decisions (*n* = 9), enabling them to make better informed decisions and/or participate more actively in future interactions with staff (*n* = 7), and providing psychoeducation for family members to, i.a., improving future interactions with staff (*n* = 3).

**Table 3:** Overview of interventions fostering family inclusion in family-staff interactions

Aim of the intervention: Study: Component(s) of interventions described in included studies:^**a**^	Creating family-staff partnerships		Family inclusion in formal decisions									Enabling family to make better informed decisions/participate more actively in future interactions with staff							Psychoeducation for family		
	1	2	3	4	5	6	7	8	9	10	11	12	13	14	15	16	17	18	19	20	21
**DIRECT FAMILY-STAFF INTERACTION**																					
Formal family-staff discussion	**+**	**+**	**+**			**+**	**+**			**+**	**+**										
**INDIRECT FAMILY-STAFF INTERACTION**																					
Family consultation/discussion by/with third party			**+**	**+**	**+**			**+**	**+**												
Staff informed about family decision				**+**	**+**			**+**													
Staff discussion with third party			**+**					**+**	**+**												
**PREPARATION/FACILITATION DISCUSSION**																					
Person who evaluates necessity of and facilitates the family-staff discussion														**+**							
Agenda setting for the discussion		**+**	**+**	**+**																	
Discussion tool for family							**+**														
Discussion tool for staff							**+**			**+**											
**FORMALIZATION OF ACTIONS/PLANS DISCUSSED**			**+**	**+**																	
**EMOTIONAL SUPPORT FAMILY**								**+**											**+**	**+**	**+**
**EDUCATION FAMILY-STAFF INTERACTIONS**																					
Family educational sessions		**+**													**+**					**+**	**+**
Staff educational sessions	**+**	**+**			**+**						**+**	**+**	**+**	**+**	**+**			**+**			
Information booklet for family	**+**			**+**									**+**	**+**							
Information booklet for staff													**+**					**+**			
**EDUCATION DEMENTIA/CARE-RELATED**																					
Family educational sessions		**+**							**+**						**+**					**+**	**+**
Staff educational sessions	**+**	**+**			**+**				**+**				**+**	**+**	**+**			**+**			
Information booklet for family	**+**			**+**	**+**								**+**	**+**		**+**	**+**				
Information booklet for staff					**+**								**+**					**+**			
Video decision-aid for family							**+**														
**PRIMARY NURSE (STAFF ASSIGNED TO FAMILY)**	**+**													**+**							
**INTEGRATED, INTERDISCIPLINARY RESIDENT ASSESSMENT AND CARE**									**+**					**+**	**+**						
**MANAGEMENT ENGAGEMENT**																		**+**			
**DEMENTIA CHAMPION**																		**+**			

^a^ Studies: 1: Bramble et al. (2011) [35]; Jablonski et al. (2005) [41]; Maas et al. (2004) [44]; 2. Robison et al. (2007) [50]; 3. Agar et al. (2017) [31]; 4. Brazil et al. (2018) [36]; 5. Kuhn et al. (2012) [43]; 6. Kellett et al. (2010) [42]; 7. Chisholm et al. (2018) [37]; Rosemond et al. (2017) [51]; 8. Reinhardt et al. (2014) [49]; 9. Saini et al. (2016) [53]; 10. Stirling et al. (2014) [56]; 11. Mariani et al. (2018) [45]; 12. Ampe et al. (2017) [32]; 13. Arcand et al. (2009) [33]; 14. Verreault et al. (2018) [59]; 15. Moore et al. (2017) [47]; 16. Van der Steen et al. (2011) [58]; Van der Steen et al. (2012) [57]; 17. Snyder et al. (2013) [54]; 18. Beer et al. (2011) [34]; 19. Davis et al. (2011) [39]; 20. Ducharme et al. (2005) [40]; 21. Paun et al. (2015) [48]

### Creating family-staff partnerships

Two different interventions aimed at creating family−staff partnerships were found. In three of the included studies, the ‘Family involvement in care (FIC)’ program [35, 41, 44] and in one the ‘Partners in Caregiving in the Special Care Unit Environment (PIC-SCU)’ [50] program were tested. Both interventions consisted of formal family−staff discussions and educational sessions for staff. In the FIC program, family and staff discussed a partnership agreement and held monthly catch-up meetings, while in the PIC-SCU a joint meeting between family, staff and the nursing home administrator was held to set goals regarding procedures and policies that affect families. To prepare the formal discussions, staff in the FIC program participated in educational sessions on dementia, the role of family in nursing homes, as well as on role negotiation and conflict resolution with family members. Family members received an information booklet on dementia and the role of family in nursing homes. In addition, a primary nurse was assigned to family members [35, 41, 44]. In the PIC-SCU program, separate educational sessions were provided for staff and family members to prepare the formal discussion. Both parties received education on dementia, family−staff communication, cultural and ethnic differences, understanding differences in values between family and staff, and on role negotiation and conflict resolution with family/staff. Prior to the actual discussion, family and staff separately set the agenda [50].

### Including family members in formal decisions

In eight of the included studies [31, 36, 37, 43, 49, 51, 53, 56], the inclusion of family members in formal decisions within the nursing homes was fostered through interventions aimed at giving family members the opportunity to decide about the residents’ end-of-life care in the nursing home. In the study by Mariani et al. [45], people with dementia and their family members were involved in care planning and the development of personalized care plans. In another study [42], the ‘Family Biography Workshop,’ in which family and staff members collaboratively develop biographies for people with dementia, was tested. Family members in included studies participated in formal discussions with direct care staff (*n* = 6) [31, 37, 42, 45, 51, 56], discussions with a palliative care team (*n* = 1) [49], and/or in discussions with an intervention coordinator/facilitator, without talking directly with direct care staff (*n* = 4) [36, 43, 49, 53]. In one study [53], family members could attend an educational session prior to the discussion. In three studies [43, 45, 53], educational sessions for staff members were provided, focusing on how to shape family−staff interactions (*n* = 2) or dementia and care-related aspects (*n* = 2). In one study, the formal family−staff discussion was facilitated by providing a discussion tool to staff (tool to aid talking about dementia and dying) [56] and in two studies to family and staff (Goals of Care video decision aid) [37, 51]. In two other studies, family members [36] or family members and staff [43] received an information booklet. In one study [49], a palliative care social worker provided emotional support to family members via phone.

### Enabling family members to make better informed decisions or participate more actively

The interventions in eight of the included studies were aimed at enabling family members to make better informed decisions and/or participate more actively in future discussions with staff [32–34, 47, 54, 57–59]. In five studies [32–34, 47, 59], educational sessions for staff members were organized, providing knowledge about how to shape family−staff interactions (*n* = 5) or about dementia or care-related aspects (e.g., end-of-life care). In one study, family members could also attend educational sessions [47]. In four studies [33, 57–59], an information booklet was provided to family members, and in one to staff members as well [33].

### Psychoeducation for family members

In three studies [39, 40, 48], psychoeducational interventions for family members were provided, consisting of emotional support (*n* = 3) [39, 40, 48] and educational sessions for family members (*n* = 2) [39, 40]. Davis et al. [39] provided the ‘Family Intervention: Telephone Tracking – Nursing Home’ (FITT-NH) aimed at improving family members’ adjustment following residents’ nursing home admission. FITT-NH consisted of emotional support for individual family members, directing them to appropriate resources within the facility, and teaching them strategies to cope with ongoing problems related to nursing home placement [39]. Another intervention, tested by Ducharme et al. [40], was the ‘Taking Care of Myself’ program, a group program aimed at empowering daughters of residents to, i.a., express their point of view to staff. In the third study [48], the Chronic Grief Management Intervention (CGMI) was tested. The CGMI is a group program for family members, aimed at providing education on dementia and on teaching skills in communication and conflict resolution with staff, and chronic grief management.

### Components of interventions to foster family−resident or family−family engagement

We identified six studies in which five different interventions contributing to family*−*resident or family*−*family engagement were tested [38, 40, 42, 46, 52, 55]. The ‘Taking Care of Myself Program’ tested in the study by Ducharme et al. [40], was – besides empowering daughters to express their view to staff members – also aimed at empowering them to feel at ease with their resident, improving their visits. In the ‘Family Biography Workshop’ described by Kellett et al. [42], family members were also invited to share the biographical materials with their relatives, thus getting involved with the person with dementia. Dassa [38] tested a program in which family members created an individualized database with personal music and photos representing life episodes of their relative with dementia. The idea was that family members could use these personal music and photos to communicate more easily with their relative, leading to an alleviation of family caregiver burden during nursing home visits. In the study by Rosen et al. [52], a prototype of a web-based educational program for family members was tested. Family members received online education on dementia and residents’ behavioral disturbances related to dementia, and on how to communicate with a resident with dementia [52]. In the studies by McNiel & Westphal [46] and Stacpoole et al. [55], the Namaste Care program was tested, a program with sensory, psychosocial and spiritual components intended to enhance quality of life and quality of care for people with advanced dementia. In the two studies, one of the key elements of the Namaste Care program was a meeting with residents’ family members and friends, exploring residents’ sources of comfort and pleasure to create an individual sensory biography of the resident. Besides contributing to the wellbeing of the resident, the Namaste Care room provides opportunities for family*−*resident or family*−*family engagement [46].

### Evaluation of interventions

Overall, few studies assessed whether or not the described intervention led to an increase in family inclusion within the nursing home. For example, in the studies aimed at including family members in formal decisions, it was not always assessed whether, due to the intervention, family members had been more often involved in decision-making. In addition, two different approaches were used for the evaluation of interventions, i.e., assessing the effectiveness of interventions or qualitatively evaluating the experiences of participants, both considering a variety of outcome measures at the family, staff or resident level. Even when different studies assessed the same intervention, the outcome measures differed. For example, in the studies describing interventions aimed at increasing family−staff partnerships, a variety of effects on family, staff members or people with dementia as well as experiences from family and staff members were considered. Overall, the four studies did not find promising effects for family−staff partnerships. In two studies testing FIC [35, 44], even negative effects for staff (e.g., an increase in role stress and role strain) and family members (e.g., decreased satisfaction with care) were found. Although for the PIC-SCU program [50] small improvements for some outcomes were found, the improvements were not sustained long-term, while participating family and staff members were very positive about the program and would recommend it to others. In some studies, it was difficult to test the effectiveness due to small sample sizes. For one study, the authors themselves indicated that the study was underpowered [31]. In another study [43], the intervention was pilot-tested in two wards, with promising effects for one ward only. In general, it can be concluded that little can be said about the effectiveness of interventions in increasing family inclusion in nursing homes for people with dementia, while the experiences of participating family and staff members were positive. Even in the absence of statistically significant effects, most family members were satisfied with the interventions. In some studies, the effects on or experiences of spouses and children of people with dementia differed.

## Discussion

From the broad palette of interventions (*n* = 24) to foster the inclusion of family members of people with dementia within the nursing home setting identified in this literature study, most were aimed at fostering family inclusion in family−staff interactions (*n* = 21), while little attention was paid to family−resident or family−family engagement within the nursing home community (*n* = 6). Only two of the 21 interventions (FIC, PIC-SCU) [35, 41, 44, 50] were targeted at creating partnerships between family and staff members. The other 19 family−staff interaction interventions focused on including family members in formal decisions (*n* = 9), enabling them to make better informed decisions and/or participate more actively in future interactions with staff (*n* = 7), or providing psychoeducation for family members to, i.a., improve future interactions with staff (*n* = 3). Nevertheless, based on the number of studies included in this review (*n* = 29), it can be concluded that the number of studies of interventions to foster family involvement in nursing homes for people with dementia is increasing. At the same time, the interventions often seem to focus predominantly on staff members. In general, it is difficult to conclude whether or not the included interventions led to an actual increase in family inclusion within the nursing home.

With the aim to contribute to the development of future interventions to involve family members of people with dementia within the nursing home setting, we had a closer look at the content of included interventions. The citizen participation ladder of Edelenbos & Klijn [23] enables us to broadly map the degree and type of family inclusion within the included interventions. In most included interventions, family members’ role can be classified as ‘informing’ or ‘consulting.’ Interventions in which family members received an information booklet only [33, 57–59], are examples of ‘informing’ family members. The PIC-SCU [50] program is the only intervention aimed at ‘co-deciding’ between family and staff members. The FIC-program [35, 41, 44] does not convincingly draw towards building a partnership between family and staff members, as it focuses more on family members and how to maximize their contribution within the nursing home. Within the family−staff interaction interventions, family and staff members are often not treated equally, hampering mutual exchange and reciprocity between staff and family members. Especially with regard to education, educational sessions are often solely organized for staff members [33–35, 41, 43–45, 59], while family members receive an information booklet only. In the three psychoeducational interventions for family members, a role for staff members is lacking completely [39, 40, 48]. Although an educational component is part of many of the included interventions, the content of education differs across interventions. While a number of interventions pay attention to education regarding family−staff interactions (e.g., how to shape the relationship), other interventions focus more on education about dementia or care-related aspects (e.g., medical decisions at the end of life).

### Implications for practice and future interventions

As equal involvement of family members in nursing homes seems complex, nursing home organizations might benefit from investing more time and money to foster family inclusion within the nursing home. Based on the findings of this literature study, the following recommendations can be made:

### Pay more attention to mutual exchange and reciprocity between family and staff members

The PIC-SCU program was the only program aimed at ‘co-deciding’ between family and staff members [50]. In this program, family and staff members were treated as equal, while in other studies, often more attention was paid to staff members. To foster mutual exchange and reciprocity, for example, more attention could be paid to two-way communication between family and staff members. In addition, if educational sessions for staff are organized, the participation of family members should be considered as well. When considering the citizen participation ladder of Edelenbos & Klijn [23], current interventions focus too much on one-way ‘informing’ or ‘consulting.’

### Consider a broader role for family members of people with dementia in nursing homes

The included interventions aimed at fostering the inclusion of family members in formal decisions focus nearly solely on decisions about the residents’ end-of-life care in the nursing home, with only two interventions involving family members and people with dementia in the broader care planning and development of personalized care plans or the development of a resident biography [42, 45]. Family inclusion refers to creating democratic engagement of families by providing them with opportunities and resources that empower them to actively participate in their relatives’ life as well as in the nursing home as a community [6]. Therefore, focusing on family members’ decision-making on residents’ end-of-life care is too limited.

In addition, more attention should be paid to interventions that contribute to family−resident or family−family engagement, as prior research has indicated that family members find it difficult to interact with their relative with dementia [60]. Moreover, to our knowledge, stimulating collaboration between family members from different relatives receives little attention.

### Provide more (formal) opportunities for family−staff discussions

In only seven of the 21 family−staff interaction interventions do actual discussions between family and staff members take place (see Table 3). Prior research indicated that a lack of formalized opportunities for families to talk to staff members and participate in decision-making can ultimately result in miscommunication between both parties [6, 9, 61]. Also, in times of high workloads, communicating with family members, especially those perceived as ‘difficult’ or ‘demanding,’ might not be a priority of staff members [27, 62]. In addition, prior research indicated that family members often find it difficult to talk to staff members too [63]. Without formal opportunities to talk to staff members, it might be the case that only the more dominant family members take their opportunity to talk to staff.

### Consider the role of an independent discussion leader/facilitator

In four of the included interventions, an independent facilitator led the discussions with family members, so that family members did not talk directly with direct care staff members [36, 43, 49, 53]. Installing a third party to collaborate with family members might be a good starting point to gain family members’ trust and to make it more easy for them to raise their voices, as family members may experience fear of speaking up for their relatives [6]. An independent discussion leader or facilitator might be installed to make sure that an actual dialogue between family and staff members takes place and could translate and explain the medical jargon often used by staff members. This could be a staff member who is not involved in the care of the residents, or a person who is not involved in the care organization (such as a counselor).

### Provide more education opportunities for family and staff members

As equal involvement of family members in nursing homes is complex and seems to be absent in most nursing homes, it might be wise to educate family and staff members more about the possible roles of the other and on how to shape equal relationships with each other. Qualitative studies on building relationships between family members of people with dementia and staff in nursing homes [40, 64–66] may give an indication of what might be important elements of future educational interventions in family−staff relationships. For example, it seems that good communication about differences in family and nurse expectations of ‘good care’ can be considered a necessity to prevent conflicts [66], highlighting the need for communication skills training. Other staff behaviors and characteristics that were associated with smoother family−staff relationships were: providing family with information by initiating a dialogue; answering families’ questions or sharing private information (e.g., staff sharing information about their family) [64, 65]; having strong interpersonal skills (e.g., being emphatic, communicating in a non-offending way, showing interest toward the resident) [65, 67]; valuing family perceptions and expertise [66]; being responsive to family concerns [67]; allowing family to be involved in care and to collaborate with staff [65, 67]; being transparent in the event of incidents or accidents [67]; and being in the same age range as the family members [66]. Identified strategies that family members might use to improve family−staff relationships were being open to staff (e.g., sharing demographic or care-related information) [64], offering help to or showing willingness to collaborate with staff members [64, 67], making emotional adjustments (e.g., looking at the situation from each other’s perspectives) [64], and using a ‘diplomatic’ communication style [67].

At the same time, in the case of people with dementia, the dementia disease has an influence on the type of relationship that is developed between family and staff members. First, because the role of family members of people with dementia in nursing homes differs from the role of family members of people without dementia [11]. Second, because family members of people with dementia often struggle with understanding dementia and its consequences for their relative, which might lead to misunderstandings with staff [66]. Therefore, family members might benefit from receiving education about the dementia disease and its consequences for their relative [68]. Prior research has indicated that staff members might also benefit from gaining more knowledge about dementia, how to care for people with dementia [69], and how to involve their family members.

### Pay ongoing attention to the specific characteristics, abilities, wishes and needs of family members of people with dementia

As in some of the included studies the effects on or experiences of spouses and children of people with dementia differed, it might be wise to target the interventions more specifically to the needs and different characteristics of family members. Contextual factors like geographic proximity, the employment status or family members’ own health status may have an impact on the role family members want to or can play in nursing homes [6]. As it is known that family members’ roles continue once a person with dementia enters a nursing home [4–6], it is notable that only one of the included interventions [39] is targeted at family members whose relative recently entered the nursing home, delivering emotional support for family members, directing them towards appropriate resources in the facility, and teaching them to cope with ongoing problems related to the nursing home placement. Investing in the wellbeing of family members whose relatives have recently entered the nursing home might contribute to a smoother development of family−staff relationships from the beginning, as family members might feel more understood instead of left alone with complex emotions [6, 19]. In addition, as nursing home settings are characterized by dying and the death of residents, it is important to continue paying attention to the emotions of family members the whole time their relative with dementia is living in the nursing home, as these emotions can shape the role a family wants to play within the nursing home [6, 70, 71].

### Implications for future research on the effectiveness of and experiences with interventions

Future studies in which interventions are tested should pay more attention to the assessment of whether or not the intervention led to an actual increase in family inclusion within the nursing home. In addition, they should carefully think about whether the components of the intervention are targeted at reaching the aim of the intervention. For example, if the intervention is aimed at reaching an equal partnership between family and staff members, it should consist of components that foster mutual exchange and reciprocity among both groups. Prior intervention research conducted in the nursing home setting (e.g, interventions to reduce the use of physical restraints), indicates that multicomponent interventions consisting of education, coaching and policy components might be more effective than single-component interventions that solely focus on education [72–74]. The fact that, based on the included studies, no firm conclusions can be drawn on the interventions’ effectiveness, may be an indication of how difficult the evaluation is. Before implementing a new intervention, researchers should define adequate sample sizes and carefully select research designs and outcome measures.

Ideally, not only family members but also the people with dementia themselves and staff members should benefit from the intervention [1]. Therefore, resident and staff outcome measures should be considered as well. While it is difficult to include the voice of people with dementia, a better evaluation of care from their perspective is necessary to deliver care that meets their needs more effectively [75]. Besides assessing the effectiveness of interventions, the experiences of participating family, staff members, and, if possible, people with dementia should also be considered. The recently developed INDividually Experienced QUAlity of Long-term care (INDEXQUAL) framework [76] could serve as a framework for developing new methods to assess experienced quality of care within the relationships of care recipient (person with dementia), professional caregiver (staff member) and informal caregiver (family member). The INDEXQUAL could also be used to assess whether or not and how the recipient, professional caregivers and informal caregivers have the feeling that family inclusion has been improved. As in some of the included studies the experiences of spouses and children of people with dementia differed, it might be wise to distinguish between different subgroups when analyzing the effectiveness or interpreting the experiences for family members.

As the involvement of family members of people with dementia in nursing homes is shaped by changes in their relationships (e.g., with the person with dementia or staff members) and roles over time, a participatory action research design with a continuing cyclical process of phases of ‘observing,’ ‘reflecting,’ ‘planning,’ and ‘acting’ might be applied for the evaluation of interventions [77]. Since, up until now, little is known on promising components of interventions to foster family inclusion in nursing homes for people with dementia, applying a participatory action research design can enable researchers to first test the effects of or experiences with individual components (e.g., ‘educational sessions,’ ‘formal family−staff discussion’) of interventions separately. Promising individual components might later be combined in a multicomponent intervention.

### Strengths and limitations of the review

This review is the first to provide an extensive overview of interventions to foster the inclusion of family members of people with dementia living in nursing homes. Two members of the research team carefully screened all potential relevant publications based on prior defined inclusion criteria. As we considered only articles that were written in English, Dutch or German, potentially relevant articles written in another language may have been missed. Furthermore, studies might have been missed that used other terms to express family inclusion in nursing homes, as a large variety of terminology exists. In addition, our search was limited to interventions reported in scientific articles. Potential interventions published in grey literature only might have been missed. Due to the heterogeneity in study designs, it was not feasible to conduct a meta-analysis. As we were predominantly interested in the components of interventions, no quality assessments of included studies were conducted. This is especially relevant for the interpretation of results. Nevertheless, the findings of included studies were summarized systematically. This was done independently by two members of the research team and discussed within the whole research team. At the same time, a general weakness of literature reviews on existing interventions is that, often, the interventions are not described in detail. A consultation of the first authors of included studies may have enabled us to describe the components of interventions in more detail. While, in this review, we focus on formal interventions, there are many informal ways in which family members can be involved in the nursing nursing home, e.g., in informal conversations with staff. Ideally, staff members should be ‘open’ for these informal quotes and expressions.

## Conclusions

Very few interventions exist that try to enhance an equal partnership between family and staff in nursing homes. Future interventions should pay specific attention to mutual exchange and reciprocity between family and staff members, to enable individual and tailored support for residents that are highly dependent on their social network. As little is known about promising (components of) interventions to foster family inclusion in nursing homes for people with dementia, more effectiveness research is needed.

## Data Availability

Database search strategies available upon request.

## Abbreviations

FIC: Family involvement in care program
PIC-SCU: Partners in Caregiving in the Special Care Unit Environment
